# Photothermal incubation of red blood cells by laser for rapid pre-transfusion blood group typing

**DOI:** 10.1038/s41598-019-47646-y

**Published:** 2019-08-02

**Authors:** Clare A. Manderson, Heather McLiesh, Rodrigo Curvello, Rico F. Tabor, Jim Manolios, Gil Garnier

**Affiliations:** 1 Department of Chemical Engineering, Bioresource Processing Research Institute of Australia, Monash University, Clayton, VIC 3800 Australia; 2 School of Chemistry, Monash University, Clayton, VIC 3800 Australia; 3 Haemokinesis Pty Ltd, Hallam, VIC 3803 Australia

**Keywords:** Lasers, LEDs and light sources, Laboratory techniques and procedures, Biomedical engineering

## Abstract

Safe blood transfusion requires compatibility testing of donor and recipient to prevent potentially fatal transfusion reactions. Detection of immunoglobulin G (IgG) antibodies requires incubation at 37 °C, often for up to 15 minutes. Current incubation technology predominantly relies on slow thermal-gradient dependent conduction. Here, we present rapid optical heating via laser, where targeted illumination of a blood-antibody sample in a diagnostic gel card is converted into heat, via photothermal absorption. Our laser-incubator heats the 75 µL blood-antibody sample to 37 °C in under 30 seconds. We show that red blood cells act as photothermal agents under near-infrared laser incubation, triggering rapid antigen-antibody binding. We detect no significant damage to the cells or antibodies for laser incubations of up to fifteen minutes. We demonstrate laser-incubated immunohaematological testing to be both faster and more sensitive than current best practice — with clearly positive results seen from laser incubations of just 40 seconds.

## Introduction

Blood transfusion is a critical treatment for a variety of haematological conditions, including cancer chemotherapy (1.7 million new patients/year in US), sickle cell disease treatments (100,000 patients/year in US), bleeding trauma, including childbirth (4 million births/year in US), and major surgery. Over 21 million blood components are transfused in the US alone every year; each having the potential for fatal haemolytic transfusion reactions if recipient and donor are not accurately matched. Furthermore, pregnant women often have a different blood group to that of their unborn child whereby blood-borne antibodies capable of crossing the placenta can cause haemolytic disease of the foetus and newborn (HDFN). Immunohaematological tests, where blood group is *typed* (determined) and antibodies are screened for and identified, must be performed for all patients, donors and pregnant mothers to prevent possible fatalities. Blood group type is based on the presence of antigens on the surface of the red blood cell (RBC) membranes, consisting of proteins, glycoproteins, glycophorins, glycolipids and polysaccharide macromolecules, forming over 346 known blood groups^1^. For each, specific antibodies can be present in a person’s plasma. This presents an enormous immunohaematological industry with hundreds of millions of tests performed globally each year.

Identification of antigens and antibodies often requires incubation at human body temperature of 37 °C. Depending on the method, this incubation step can take 5 to 30 minutes. This delay adds to pathology cost and turnaround time and can endanger a patient’s life. Current incubation methods consist of dry-air incubators (ovens), heating blocks or hot water baths which all rely on the relatively slow thermal energy transfer methods of conduction and convection. Electromagnetic radiation-based blood warming technologies using radio- and microwaves have been used since the 1960s in pre-transfusion blood warming for the prevention of patient hypothermia^2,3^. However, these techniques have lacked the very precise temperature control or fast and uniform heating rates required for sensitive immunohaematological reactions. Furthermore, for use with the highly sensitive gel card diagnostics^4^, microwave incubation which lacks targeted and localised heating, would heat the whole gel card, potentially damaging the gel matrix, making it an unsuitable method for immunohaematological tests.

Optical heating via laser-light incubation provides an opportunity to not only rapidly heat directly inside the sample volume, but also to preferentially heat the surface of the red blood cell (erythrocyte) and activate antigen-antibody binding. Here, we present laser-based incubation for the photothermal heating of red blood cell and antibody samples in traditional gel cards, where the optical absorption properties of blood and water produce thermal energy (heat) via non-radiative decay processes. By selectively controlling the power, wavelength and positioning of the laser light, the incubation time can be considerably reduced without significant damage to the cells or biomolecules.

Light-to-heat converters have been explored in biomedicine for a range of applications, including modern therapies, imaging and biosensing^5,6^. Infrared lasers have also been used for heating of 50 µL droplets in polymerase chain reaction (PCR) studies^7,8^ where in our study, the RBCs may act as photothermal agents.

**Figure 1:**
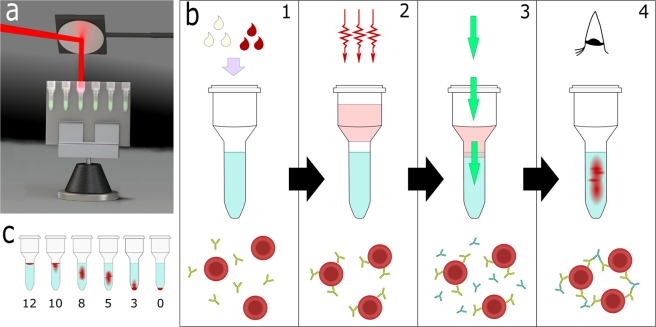
Experimental laser chamber and gel card indirect antiglobulin test (IAT). (**a)** Our laser incubation chamber illuminates a blood-typing gel card column containing a red blood cell (RBC)-antibody suspension with infrared laser light (980 nm). **(b**) Experiment steps: (1) RBC and antibody solutions are added to the gel card upper chamber. (2) RBC-antibody suspension is incubated by laser photons entering from above. (3) Gel card is centrifuged to mix the antibody-bound or unbound RBCs with the anti-antibody (IgG) and pass through the gel column. (4) Agglutinates and RBC positions are observed. **(c)** Scoring the result: positions of the agglutinates indicate strength of result.

Immunohaematological tests use the specific binding (complexing) of antibody to antigen (epitope) to form RBC agglutinates (cell aggregates) to indicate a positive result. Immunoglobulin M (IgM) antibodies are pentamers and are able to bridge the RBC’s native repulsion charge to form agglutinates directly; and are used for the detection of most antigens. However, the monomer immunoglobulin G (IgG) antibodies, whose presence must be detected in pregnant mother or patient plasma, cannot directly agglutinate cells. They require secondary anti-IgG antibodies to bridge the IgG sensitized cells — forming the indirect antiglobulin test (IAT) or Coombs test^9,10^ (Fig. 1b). Sensitization of ‘warm reacting’ IgG antibodies occurs best at 37 °C, human body temperature, requiring a minimum 5 to 15 minute incubation, with longer incubation periods often enhancing the reaction and reducing the so-called ‘cold reacting’ IgM antibodies from returning false positives^11–13^.

In this proof of concept study, we explore the roles of incubation time and temperature of the Rh blood group system’s D antigen and the IgG anti-D antibody. It is the presence of the RhD epitope, a multipass transmembrane protein, which indicates the positive or negative attributed to a person’s ABO blood group type. Anti-D is most commonly present as IgG in a person’s plasma. It is the most important type of antibody to test for in transfusion medicine, and is the biggest cause of HDFN.^14,15^. Complexation of the epitope (antigen) and paratope (antibody binding site) has a temperature dependent binding rate however the literature is lacking compelling evidence and the issue has not been investigated in decades^11–13,16–18^. This provided an opportunity to enhance the current standards of immunohaematological testing with contemporary science.

Here, we investigate the potential of laser incubation as a rapid and sensitive method for immunohaematology. We optimised a laser incubation system and determined the parameters required for effectively and stable heating the RBC-antibody solution. The role of incubation time and temperature are demonstrated to play key roles in strength of result. Furthermore, laser incubation is shown to both enhance agglutination strength for weak antibody solutions and reduce required incubation time for successful positive results. We show that the laser preferentially heats the RBC surface’s micro-environment, inducing RBC-antibody complexation, via the mechanisms of infrared light absorption and scattering by RBC solutions. Finally, we demonstrate that the laser does not induce RBC damage for incubations of 15 minutes or less and that antibody potency is preserved for up to 30 minutes in this optimised system.

## Results and Discussion

### Laser-incubation chamber and gel card diagnostics

A laser incubation chamber was designed and built to heat a RBC-antibody sample in a standard immunohaematological gel card diagnostic^4^ via infrared laser illumination (Fig. 1a). The RBC and antibody solutions are applied to the upper chamber of a gel card column for incubation (Fig. 1b.1). Continuous wave (CW) infrared (980 nm, 2 W) laser light is directed down through the open top of a gel card well containing the 75 µL RBC-antibody suspension (Fig. 1b.2). The sample absorbs the laser light, converting it into thermal energy which is then dispersed through the volume via conductive and convective processes. The antigens on the surface of the RBC and the antibodies in the local micro-environment are heated, activating the binding sites for complexation. After an incubation (heating) period, centrifugation draws the IgG sensitized RBCs into the anti-IgG solution and into the gel column (Fig. 1b.3). Agglutinates are formed for a positive test and are trapped at varying levels within the gel column (Fig. 1b.4).

As is standard for this test, the final position of the RBCs in the gel column is used to determine if the test is positive, and to what strength — described by a score from 0 to 12 (Fig. 1c)^19^. The strongest positive is scored as 12, and weaker positives are scored in integers down to 1 depending on the relative height of the agglutinates. Unagglutinated individual RBCs (negative result) pass entirely through the column and are scored as 0. A higher score can indicate the presence of more antigens on the RBCs, a higher antibody concentration or a stronger binding affinity.

### The benefits of laser incubation

When Coombs first developed the IAT in the 1940s, incubations of 30 minutes at 37 °C were the standard. In the decades since, this incubation time has been greatly reduced to the current fastest method of a five minute incubation, using the gel card system as selected in this study (Stargel_10_, Haemokinesis) which incubates in a gel card heating block (37 °C). We present a direct comparison of this heating block to laser (36–38 °C) and room temperature (22–24 °C) incubations for times of 0–30 minutes to explore the roles of temperature and time.

For each temperature and time point, a full titre (two-fold serial doubling dilution) of a concentrated antibody solution was performed, with each dilution incubated separately in a gel card. The titre produces a series of antibody solutions, each with progressively halved concentrations of antibody. Testing each dilution separately identifies the dilution factor with the weakest concentration (*titre number*) to which the test conditions are sensitive.

**Figure 2:**
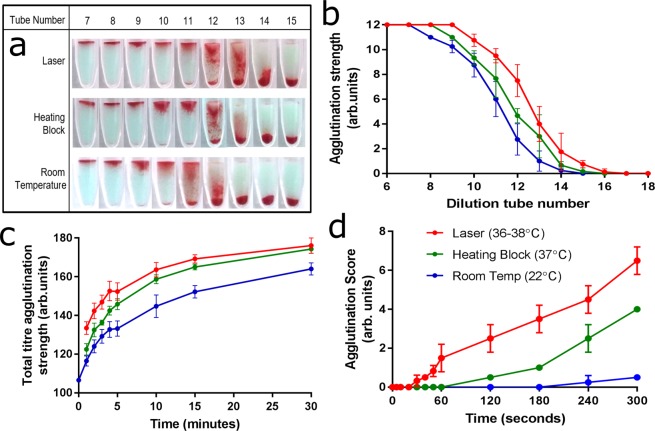
Laser incubation gives stronger results faster. Comparison of incubation methods. Incubation at room temperature (22–24 °C) is compared to two methods of heating: heating block (37 °C) and laser illumination (36–38 °C). **(a)** For a two-minute incubation time, subsequent dilutions of antibody show positive results (left) and negative results (right). Laser incubation gives clearer and more consistent positive results for weaker antibody solutions than the other two methods (a higher titre number). **(b)** Scoring each dilution out of 12 allows plotting of the results. **(c)** Summing the score across the full dilution gives a total score. The role of incubation temperature and time are explored. **(d)** Sensitivity control check using 0.05 IU/mL antibody.

Post-incubation, the card is centrifuged, then imaged and the position of the RBCs in the gel column is observed. Figure 2a presents results for the 2-minute incubation time-points comparing each method. For both laser and heating block incubations at the higher temperatures, positive results are seen for weaker dilutions of antibody which are negative (or close to) for room temperature (Tube Numbers 13 to 15), giving a higher titre number. With laser incubation, this advantage is furthered still, resulting in nearly a two-tube titre number increase (i.e. 4 times less antibody) compared to room temperature. Figure 2b presents the scores attributed to the dilutions in Fig. 2a.

The total agglutination strength for each dilution series is calculated by adding the score for every tube in the serial dilution, giving a total agglutination strength score for each time and temperature combination (Fig. 2c). Scoring a titre solely by its titre number is common. However, summing the agglutination strength score for every dilution for the full titre has benefits. A high-titre-low-affinity antibody would have high titre number despite having a relatively weaker antibody and is described more accurately by the summed score.

An immediate spin test (0 minutes incubation, room temperature (22–24 °C)) gave a positive agglutination result, with a titre number of 1024. The effect of increased incubation time is clear, where all three methods have inverted exponential-like increase. After 15 minutes, enhancement from further incubation is minimal seeming to plateau to a maximum just beyond the 30-minute mark. The role of time is evident: longer incubations at room temperature give stronger results than shorter incubations at increased temperature. The most significant increase occurs in the first five minutes. The advantage of increased temperature is also clear, with both heating methods significantly better than room temperature. For obtaining strong results in a short time, heat plays a critical role.

Laser incubation gives the strongest result. However, by the 30-minute mark this advantage is lost, confirming the critical role of the initial heating period. The laser incubation at 2 minutes gives a comparable result to the current fastest method of 5 minutes with the heating block. This represents a greater than 50% reduction in incubation time. If heat is transferred to the antigen-antibody binding site directly and rapidly, complexation forms quickly and a positive agglutination reaction is achieved faster.

A sensitivity control check was performed using a weak antibody solution of known strength (0.05 IU/mL) to verify that laser incubation can detect weak antibodies. Figure 2d presents the results for incubations of 5 seconds up to 5 minutes. The laser-incubated tests give consistent positive results at 40 seconds when the other incubations methods indicate a negative. The heating block incubated test requires 3–4 minutes to achieve the same sensitivity as what the laser incubation can achieve in less than one minute.

### The optics of laser heating and temperature measurement

**Figure 3:**
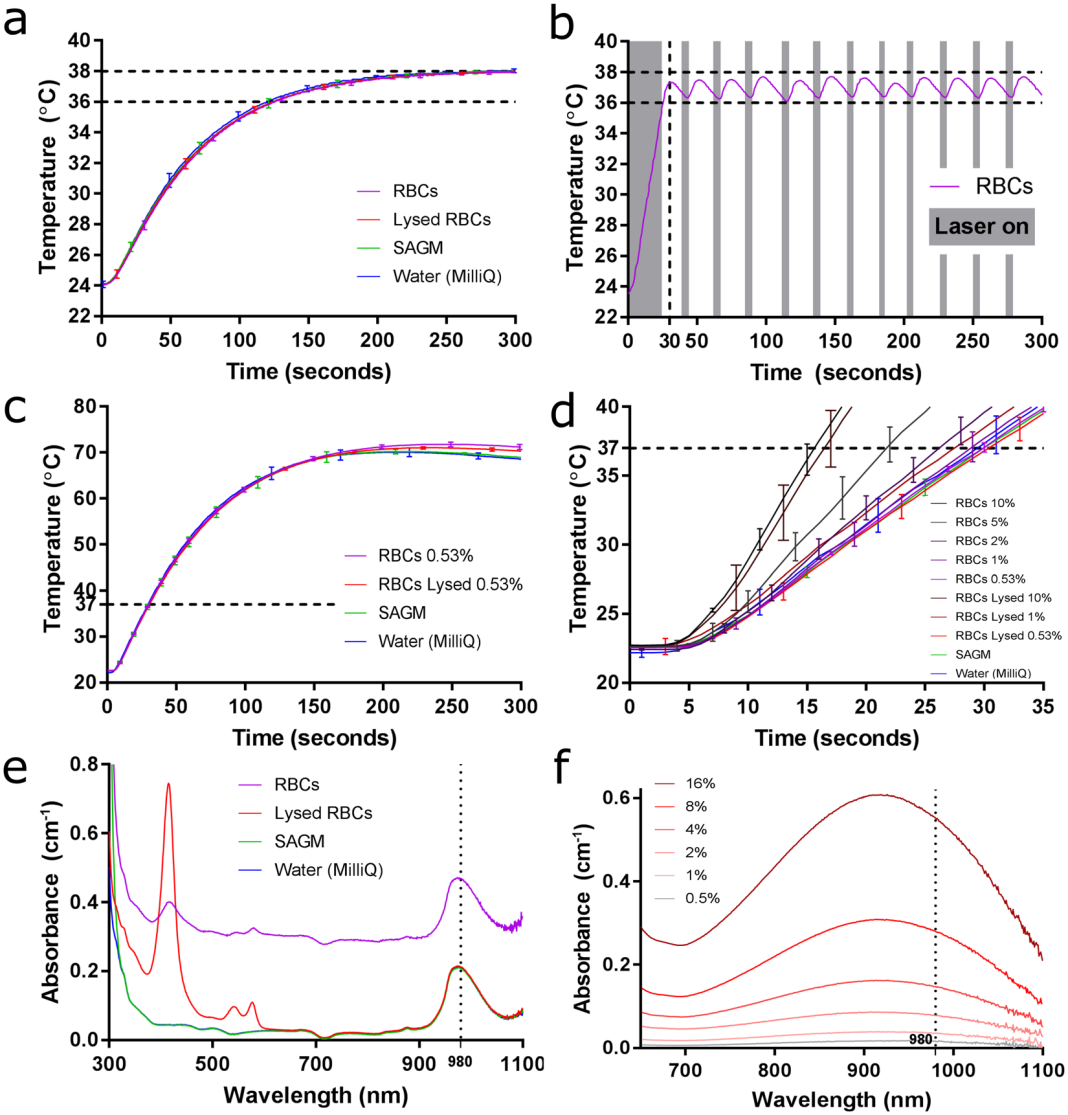
Laser light absorption by RBCs. (a) Heating block incubation heats water, whole and lysed RBC suspensions (75 µL) at the same rate, reaching 37 °C in 150 seconds. (**b**,**c**) Laser illumination rapidly heats sample volumes (75 µL), reaching 37 °C in under 30 seconds. (**b)** Laser illumination is pulsed on (grey) and off (white) to heat and maintain sample temperature between 36 °C and 38 °C. **(c)** Without pulsing, the sample will heat to 70 °C. **(d)** Increasing the concentration of both whole and lysed RBCs increases the heating rate. **(e)** Absorption spectra for RBCs and water across the visible and near-infrared wavelength ranges. **(f)** Absorption of lysed RBC suspensions for various concentrations around 980 nm (water baseline absorption subtracted).

Traditional incubators, such as heating blocks, dry air ovens and water baths, rely on conductive and convective processes to transfer heat energy from the outside of the sample volume to the inside. Although thermal conduction through plastic and water is on the microsecond (10^−6^ s) timescale, the heating rate of the sample in such incubators is slowed by a strong dependency on the temperature gradient between the heater outside the gel card (necessarily at 37 °C) and the sample. Increasing the incubator temperature would hasten the heating, but control of the sample to be kept within range (36–38 °C) would be lost. Also, samples containing red blood cells necessarily heat at the same rate as their water-based solute (Fig. 3a) taking around 150 seconds to reach 37 °C in the heating block.

Light can penetrate the centre of the sample where it is converted into thermal energy on the picosecond (10^−12^ s) timescale. The rate of temperature increase of the sample is no longer dependent on a temperature gradient with an external environment, but rather on the rate of photons (laser power) entering the sample. With a 2 W continuous wave laser with 980 nm infrared photons, a 75 µL sample with 0.53% v/v RBCs is heated to 37 °C in under 30 seconds (Fig. 3b,c) which is at least two minutes faster than the heating block. This heating rate matches theory, as follows. For water, with a specific heat capacity of 4.184 calories (J/gK), the energy, *E*, required to heat such a volume, *V*, from room temperature, 22 °C to 37 °C,*ΔT*, can be calculated: *E* = *cVΔT*= 4.707 J. For a laser power, *P*, of 2 J/s, reflected off a mirror with 96.9% efficiency (at 980 nm), the heating time, *t* = *E/P* = 2.4 seconds. The absorption efficiency of water at 980 nm is measured to be 0.203 cm^−1^ (Fig. 3e), that is 20.3% of the light is absorbed every 1 cm travel through the water. So for a 5 mm gel card chamber depth, the time is *t* = 2.4/0.1 = 24 seconds, matching what we see experimentally, with other minor reflections and losses not taken into account. This indicates a very high photothermal conversion efficiency of the water and RBCs.

Uninterrupted laser illumination heats the sample to high temperatures — around 70 °C for this system — until equilibrated by cooling processes (thermal emission, evaporation, etc) (Fig. 3c). Samples heated to this high temperature demonstrated significant RBC damage (dark colouring and coagulation evident) and some evaporation of the solution was evident.

To control the temperature of the sample to within the 36–38 °C range, the laser incubation chamber incorporates a temperature control feedback system: the laser is turned on and off by opening and closing a shutter in response to the temperature detected by an infrared thermometer. The temperature profile and laser on/off cycling of a typical 5-minute laser incubation is shown in Fig. 3b. The duty cycle is 4.5/24 seconds, meaning that the laser is on for just under 20% of the time. The maximum temperature reached in this example was 37.71 °C with an average of 37.03 °C (measured from the when 36 °C was first reached until the 5-minute conclusion). The laser incubation method is therefore highly stable and sensitive.

The time taken to reach 37 °C with laser incubation is just under 30 seconds compared to the heating block of 150 seconds (Fig. 3a–c). This corresponds to the 2–3 minute benefit of laser incubation. Furthermore, enhanced reaction strengths are seen for 1-minute incubations, and under one-minute incubations with the sensitivity control check. Rapid incubation to 37 °C has a distinct benefit.

The laser may also be preferentially heating the RBC surface, hastening the temperature to 37 °C faster than what we measure. To address this, we must first understand what temperature we are measuring in our system. The infrared thermometer used in this study cannot detect the temperature of RBC surface alone. Objects at human body temperature (37 °C), T, emit infrared light over a range of wavelengths, with a peak wavelength, λ_p,_ at around 9.34 µm — a relationship governed by Wien’s displacement law, *λ*_*p*_*T* = 2.898 × 10^−3^ mK. As the sample is heated by the laser from 23 °C to 38 °C it is the peak wavelength of the emitted thermal radiation which is detected, shifting from around 9.8 to 9.3 µm. Sensitive measurement of the wavelengths of the emitted photons allows remote monitoring of temperature without the risk of interference or thermal losses via thermocoupling. But this system can only measure the bulk solution properties and not the temperature of the RBC surface directly.

We see that increasing the concentration of both whole and lysed RBCs increases the rate of temperature increase by the laser (Fig. 3c). Blood heats faster than water. Optical spectroscopy provides an insight into the optics of an RBC solution to explain why. In the visible region of the spectrum (around 400–780 nm) blood and water respond very differently (Fig. 3d): haemoglobin has strong absorption peaks in the blue (420 nm: Soret band) and green (540 nm & 575 nm: oxyhaemoglobin β- and α-bands, respectively) giving blood its distinct and intense red colour; whereas water has no absorption peaks in this region, making it transparent. In the near-infrared region (NIR) both haemoglobin and water have absorption peaks: haemoglobin near 914 nm (Fig. 3e) and water near the illuminating wavelength of 980 nm (Fig. 3d). For water, this absorption is known to correlate with non-radiative vibrational energy relaxation modes of the H_2_O molecule — creating heat.

Illuminating RBCs in an aqueous solution with near infrared radiation creates heat mainly via interaction with water but this is enhanced by the optical properties of RBCs, particularly the absorption of haemoglobin — indicating a preferential heating of the surface of the RBC. Trapping RBCs by NIR optical tweezers has been shown to heat the RBC more than the surrounding water^20^. Total internal reflection of the RBC may lead to further absorption and heating^21^. Scattering of the whole RBCs^22^, indicated in the absorption spectrum in Fig. 3d by the vertical shift up and a peak reduction of the data, will enhance the heating effect further due to the multiple passes of the photons through the sample, increasing the likelihood of absorption. The RBCs are acting as photothermal agents. The extra heat (beyond that of the water absorption) at the RBC surface rapidly heats the surrounding micro-environment, where interaction of the antigen with antibody’s paratope occurs.

This extra thermal energy generated by the photothermal activity of the RBC then dissipates through the bulk solution via convective and conductive processes further heating the bulk water matrix. The first few seconds of the Fig. 3d indicate little temperature rise, although the laser is on. This represents the finite time taken for the light to be scattered, absorbed and re-emitted a multitude of times enough to heat the RBCs and the surrounding water. The thermal emission must then similarly be scattered until reaching the infrared detector.

Performing the gel card test with a higher concentration of RBCs in the sample solution could allow heating to 37 °C faster than 30 seconds. However, there are limits in the volume allowances of current gel card technology to hold the corresponding increased antibody volume required for accurate testing. We can induce from these data that the local optical environment on the RBC surface is sensitive to infrared radiation and will be hotter than the greater water matrix. This may be the cause of the rapid increase in agglutination strength. Despite enhanced results, it is critical to determine whether the laser light is damaging the antibody or RBCs.

### Laser incubation effects on RBCs and antibody

The photothermal effects of the 980 nm laser light on human tissue can cause significant damage — sometimes to great benefit like when used for laser ablation of tissue and skin welding^23,24^. However, the effect on individual RBCs and antibodies in solution is unknown and of interest here. Pulsed laser light can cause microbubbles in cells^25,26^ and continuous wave (CW) lasers can also create localised vaporization in RBCs when in the ‘fast’ heating regime^27^. The photothermal effects of the CW laser incubation used in this study were explored. We investigated three issues: (1) Can false positive aggregation or false negative dissociation occur? (2) Can laser incubation damage antibodies? (3) Can laser incubation damage cells?

**Figure 4:**
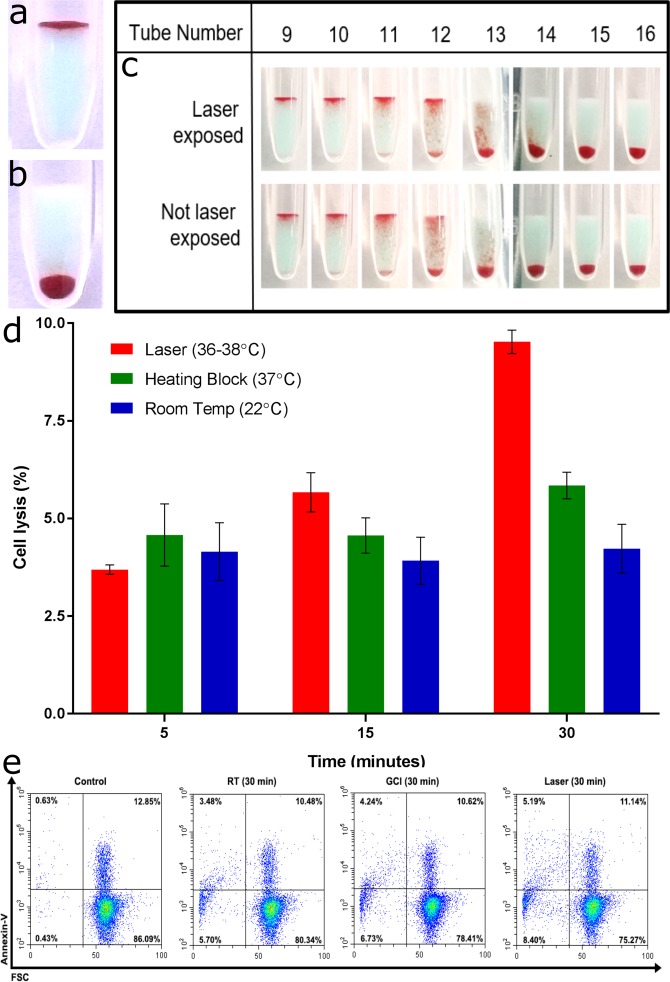
Laser damage studies on the RBCs and antibody. (**a**,**b)** Examples of typical positive (**a**) and negative (**b**) results for laser incubation: **(a)** Laser incubation gives correct positives and does not dissociate the RBC-antibody complex. **(b)** Laser incubation does not induce RBC direct agglutination or non-specific binding. **(c)** Laser-exposed and unexposed antibodies have similar potency indicating the laser does not damage the IgG biomolecules. **(d)** Flow cytometric analysis of haemolysis for different incubation times and methods. **(e)** Longer (30 minute) incubations by laser cause more RBC lysis.

Firstly, throughout our experiments we have consistently seen the strong positive results expected (Fig. 4a). The laser does not dissociate the antigen-antibody complex and we do not see false negatives. Nor have we seen false positives (Fig. 4b). The laser does not induce non-specific binding of the antibody with negative RBCs. Nor does it induce RBC aggregation or coagulation.

Secondly, to test whether the laser damages the antibody, titres of antibody solution with and without exposure to the laser light were performed. The laser-exposed antibody does not give a lower titre number, indicating no reduction of antibody number or binding strength (Fig. 4c). Rather, laser incubation is seen to consistently give a slightly stronger result in the final positive dilution — potentially indicating a long-lasting conformational change of the paratope; and requires further study. Conformation of IgG antigen-binding fragments have been shown to be thermally responsive^28^.

Thirdly, damage to the red blood cells was examined by flow cytometry where the proportion of intact cells in a sample can be counted using a fluorescent marker. Incubations of 5, 15 and 30 minutes were performed for the same three incubation methods: room temperature, heating block and the laser; and the degree of lysis (indicating dying RBCs) was measured (Fig. 4d). For 5-minute incubations, all three methods give comparable levels of lysis (around 4%). For 15 minutes, laser incubation produces a further (1–2%) degree of lysis than the other methods. The long and outdated incubation time of 30 minutes (Fig. 4d,e) showed increased lysis for all methods, significantly more by the laser (up to nearly 10% total lysis). This indicates that very long incubations by the laser are not recommended. However, it further suggests a preferential heating rate at the RBC surface.

These data correlate well with the findings of a recent review of blood warming technologies which concluded that RBC fragility was temperature rather than incubator-type dependent, where for fluid-based warmers, no cell death should occur if samples are kept below 46 °C^29^. Radio- and microwave blood warmers have been found to produce some degree of haemolysis^30^. Our study adds to this field of research that laser incubation also does not significantly damage RBCs for up to 15-minute incubations below 38 °C.

## Outlook

We have designed and built a laser-incubator which can rapidly, sensitively and reliably heat red blood cell and antibody solutions for immunohaematology tests. This was validated with the indirect agglutination test (IAT) in gel cards. Our study demonstrates that the agglutination strength of red blood cell (RBC)-antibody (IgG) complexes depends on both time and temperature, where rapid application of heat hastens and enhances test sensitivity.

Laser incubation targets the heat directly inside the sample. Our investigations of the optics of RBC solutions, by analysing the absorption and emission of infrared light, reveals that the red blood cells act as photothermal agents. The laser directly heats the surface of the RBCs, thermally activating the antigen-antibody binding needed for a positive test result. Incorporation of targeted laser incubation technology into current immunohaematological practice could include combining IgM and IgG antibody tests onto the same gel card, for simultaneous forward and reverse screening. Further, there is the potential for one-step testing where incubator and centrifuge are combined — leading to rapid high-throughput results. Increased sensitivity makes the test cheaper by requiring less antibody, but importantly may require less of the patient’s plasma — a finite resource in the pathology setting. With over 21 million blood units transfused per year in the US, laser incubation can dramatically hasten and simplify immunohaematological laboratory practices.

Laser incubation can be valuable when time and accuracy is vital. In critical and emergency settings, particularly in mass trauma events where there is not enough O-negative blood (the “universal donor”) for all, pre-transfusion testing must be performed. Requiring a full cross-match of donor and recipient blood, this testing adds significant time delay to treatment. With laser incubation’s potential for a one-minute test in a single diagnostic, pre-transfusion testing could be brought out of the pathology lab to point-of-care, further reducing the time to treatment — with the potential to save the lives of bleeding-out patients.

Expanding this study to test a range of RBC antigens and plasma-borne antibodies is critical for clinical and pathological immunohaematology. Application of laser incubation more generally to heating of blood and blood products, to rapid pre-transfusion blood warming and to serology is promising. Laser incubation is an interesting technology to further explore activation of antibody-antigen and enzyme-substrate complexes.

## Materials and Methods

### Red Blood Cell samples

De-identified human whole blood samples were provided by the Australian Red Cross Blood Service, obtained with written informed consent in accordance with the recommendations of Blood Service Human Research Ethics Committee (BSHREC). This study was carried out with approval of and in accordance with the recommendations of the Monash University Human Research Ethics Committee (MUHREC). Whole blood samples were stored (refrigeration at 4 °C) with ethylenediaminetetraacetic acid (EDTA) as anticoagulant and were used within two weeks post-collection. Reagent red blood cell solutions (Stargel_10_ 3 cell screen, Haemokinesis) were used for all RBC-antibody incubation tests: positive tests used an equal part pool of Cell I (R1^w^R1) and Cell II (R2R2) solution, and Cell 3 (rr) was used for all negative tests. Concentrated red blood cells (RBCs) were extracted and diluted to various concentrations as needed in either Milli-Q water (Direct-Q 3 UV-R, Merck) to lyse them or a hypertonic saline-adenine-glucose-mannitol (SAGM) solution (sodium chloride 8.77 g/L, dextrose 9.0 g/L, adenine 0.169 g/L, mannitol 5.25 g/L) to keep cells whole and to enhance the antigen-antibody binding strength and rate.

### Antibody dilutions

Immunoglobulin-G (IgG) anti-D antibody For Further Manufacturing Use (FFMU) (clone MCAD6, Seqirus) was used for all antibody tests except for the sensitivity control test, which used Quotient ALBAcheck Anti-D 0.05 IU/mL. Antibody titres used a serial doubling dilution of the antibody, where from the neat undiluted antibody solution (Tube Number 1), each subsequent dilution (higher Tube Numbers) reduces the concentration of antibody by a factor of two. The relative concentration of antibody in each subsequent dilution is the inverse of the Dilution Factor. The diluent was a 5% bovine serum albumin (BSA) solution (30% BSA solution (Sequiris) diluted in phosphate buffered saline (PBS) solution (0.9% g/L, pH 7.2–7.6, PBS tablets, (Sigma-Aldrich) in Milli-Q water).

### Gel card column agglutination test (CAT)

The RBC-antibody mixtures were prepared directly in the gel card wells (Stargel_10_ AHG System, Haemokinesis) with 50 µL of RBCs at 0.8% concentration and 25 µL of antibody solution, producing a final RBC concentration of 0.53%. Within 30 seconds post-incubation, centrifugation of the gel card occurred (PP-IND-001 Centrifuge where the RBC-antibody mixture mixes with the anti-IgG antibody (forming the Indirect Antiglobulin Test (IAT)) prior to reaching the gel column (bead matrix) which separates agglutinated (positive result) and individual (negative result) RBCs. Digital images (Status1, Haemokinesis) of the resultant gel card columns were recorded.

### Incubation and temperature monitoring

Room temperature experiments were performed within the temperature range of 22–24 °C in standard laboratory conditions.

The gel card heating block (Prototype #5, Haemokinesis) has a fixed temperature of 37 °C. Temperature was detected during incubation directly inside the sample volume in a gel card well using a thermocouple probe (Type K, QM1283, Jaycar Electronics) and was monitored and recorded via a thermocouple measurement device and computer (USB-TC01, National Instruments).

Laser incubation was provided by a bespoke laser incubation chamber. Infrared laser light from a 980 nm CW diode laser (Dragon Lasers, ChangChun, JiLin, China) with an output power density of 2 W/cm^2^ (measured with a integrating sphere power sensor, S145C, Thorlabs) was reflected down into the gel card well via a mirror (97% reflectance, PF10-03-P01, Thorlabs) to illuminate the RBC-antibody mixture from above (not through the gel card plastic). Temperature of the sample mixture in the gel card was monitored at 1 second intervals by a non-contact infrared thermometer (CSlaser hs LT, Optris) with a spectral range of 8–14 µm (corresponding to −20–150 °C) and was focused on the side of the gel card well (spot size 1.4 mm) at the centre of the sample volume. The temperature of the mixture was kept between 36 and 38 °C by a temperature controlled feedback system (MATLAB R2018a, Mathworks^31^): monitoring at one second intervals, the sample temperature is increased/decreased by turning the laser illumination on/off the by opening/closing (respectively) a diaphragm leaf shutter (SHB1T, Thorlabs) controlled via a 5 V input signal (Arduino Uno) driven by the feedback system program.

All experiments for monitoring temperature increase rates were recorded in triplicate with dilutions of RBCs from whole blood donations. All temperature data were recorded at 1 second intervals.

### Absorption spectroscopy

Absorption spectra of RBC solutions (from whole blood donations) were acquired using a spectrophotometer (Cary 60 UV-Vis, Agilent Technologies) over the visible and near-infrared wavelength ranges (350–1100 nm). Comparison of intact (SAGM) and lysed (Milli-Q) RBC solutions were done by creating solutions of each diluted to the same concentration of 0.0265% (1/20^th^ of the standard 0.53% concentration). Each was centrifuged for three lots of 5 minute spins (15 mins total) at 3000 rpm with the whole RBCs washed between each spin. Each spectrum was obtained four times and averaged. Spectra (650 −1100 nm) comparing different concentrations of lysed RBCs were obtained by creating a solution of 16% RBCs in Milli-Q water then serially diluting it down to 0.5%. Solutions were centrifuged for 30 minutes at 4000 rpm to remove membrane fragments to reduce scattering effects. These spectra were obtained twice each and averaged with the Milli-Q water absorption spectrum as the subtracted baseline.

### Laser Damage Studies — Antibodies

Detection of laser-induced damage to antibody was tested by measuring the serial dilution titre number of antibody solutions with and without exposure to laser light. After laser incubation for 30 minutes in gel cards without gel, the cards were allowed to cool and then spun to sediment the negative RBCs (Cell 3) and a 25 µL aliquot of antibody solution was extracted. This was transferred to 175 µL of diluent (5% BSA solution), giving a dilution factor of 8, from which a serial dilution was performed. 25 µL from each dilution was tested against a 50 µL of 0.8% pooled D positive cells (Cell 1 and 2) in AHG gel cards. These tests were incubated in the heating block for 5 minutes before being centrifuged and scored in the usual method. All tests were performed in triplicate.

### Laser Damage Studies — RBCs

Detection of laser-induced damage to RBCs was tested by flow cytometry (Cytoflex, Beckman Coulter) to detect for cell lysis (Annexin-V Apoptosis Detection Kit 1, BD Bioscience). After incubation, the Rh negative (Cell 3) RBCs and antibody suspension was gently mixed in the gel card to resuspend any settled RBCs. From this a 25 µL aliquot was taken and added to 150 µL of binding solution from the Annexin-V Apoptosis kit. This solution was left to stand for 90 minutes at constant temperature and humidity (25 °C, 50% humidity). Positive control samples with induced cell lysis were made from a relatively hypotonic solution of 85% SAGM and 15% Milli-Q water. The two negative control samples had one with FITC dye and one without. Cytometric analysis on 10,000 events per sample were recorded in triplicate.

## Data Availability

All data and materials used in the study are available.
